# Influence of Waste Toner on Asphalt Binder: Chemical and Rheological Characterization

**DOI:** 10.3390/molecules28062794

**Published:** 2023-03-20

**Authors:** Prince Igor Itoua, Daquan Sun, Ping Li, Shihui Shen

**Affiliations:** 1 Key Laboratory of Road and Traffic Engineering of Ministry of Education, Tongji University, 4800 Cao’an Road, Shanghai 201804, China; princeitoua@tongji.edu.cn (P.I.I.); dqsun@tongji.edu.cn (D.S.); 2 School of Traffic and Transportation Engineering, Changsha University & Technology, 2nd Section, Wanjiali RD (S), Changsha 410114, China; li_ping@csust.edu.cn; 3 Department of Engineering, Penn State University, Altoona, PA 16601, USA

**Keywords:** modified asphalt binder, waste toner, rheology, recycled material, microscopic test

## Abstract

The growing amount of waste toner (WT) has posed a significant environmental challenge. Meanwhile, researchers are interested in the feasibility of utilizing waste toner as an asphalt binder modifier because its primary chemical components (Styrene-acrylic copolymer and carbon black) are known to improve asphalt properties. The objective of this study was to evaluate the chemical and rheological properties of the waste-toner-modified asphalt binder and hence determine the suitability of integrating waste toner for asphalt modification. The waste-toner-modified asphalt (TMA) binders were produced by blending base asphalt with two types of waste toners of different gradation sizes. Microscopic tests such as x-ray fluorescence (XRF), attenuated total reflectance transform infrared spectroscopy (ATR-FTIR), and scanning electron microscopy with energy dispersive X-ray (SEM-EDS) and fluorescence microscope, as well as rheology tests such as multiple stress creep recovery (MSCR) tests, oscillation tests, and bending beam rheometer tests were performed. The FTIR results showed that there was a chemical reaction between waste toners and base asphalt binder. A fluorescence effect was observed on the binders produced with different toners used in this research. The binder modified with an optimal content of 8%WTs revealed better high and low-temperature properties. Additionally, 8%WTs used in this research could change the PG70-22 binder to PG76-22 binder. The rutting properties of asphalt material were improved for its improved elasticity. In addition, the 200-mesh TMA binders were desirable with respect to waste toner particle size. Overall, there is a benefit to using waste toner in the asphalt industry.

## 1. Introduction

### 1.1. Background

Recycled toner from the printing industry is disposed of with a high rate of 3000 to 4000 tons per year, which could be a toxic waste and cause environmental hazards [1] if not treated properly. Currently, the two main methods of treating waste toner materials are the incineration process and the landfill process [2]. However, some researchers argued that these methods may not be appropriate and can have a negative impact on the soil and underground water. For this reason, Tian et al. [3] worked and found that waste toner could be used as a precursor to synthesize 3D porous graphene oxide (GO)hydrogel, which is a well-known method in the material science field for recycling waste toners. Furthermore, transforming waste toners into iron by using a thermal transformation process was also considered to be a sustainable recovery technology for such material [4]. A vacuum gasification approach was also developed [5] to convert waste toner materials into valuable products [6].

Although the above techniques can be used as a pathway for overcoming the disposal problem of waste toners, the amount of toner powders to be recycled is still insignificant compared to the amount produced by the printing industry every year.

In the meantime, a few studies have been conducted on using waste toner as an asphalt binder modifier. Based on laboratory studies, Notani et al. [7] found that adding 12% of waste toner can improve the rutting resistance of asphalt binder and mixtures. This finding was confirmed by Huang et al. [8] who added that by using it as a value-added modifier in the pavement construction industry, not only were the properties of asphalt enhanced, but also the disposal waste toners were recycled in an environmental way. Moreover, the stiffness of asphalt binder increased after adding waste toner according to Yildirim et al.’s research [9]. Incorporating 10% of waste toner into asphalt binder transformed its PG from PG64-28 to PG70-22 [10].

Very few studies have been completed to investigate the effect of waste toners on the low-temperature performance of asphalt binders and the results are sometimes contradictory. Notani et al. [11] found that adding 8% of WT improved the low-temperature performance of base asphalt; however, they also pointed out that the modified binders exhibited unpleasant behavior at toner concentrations greater than 8%. In addition, the findings of Yildirim et al.’s research [12] suggested that the waste-toner-modified asphalt binder was more susceptible to low-temperature cracking. Furthermore, blending waste toner into asphalt shows an improvement in the number of loading cycles, shortening the crack length and degrading dissipated energy, resulting in increased fatigue resistance of asphalt binders [13]. In contrast, Showkat et al.’s work reported that there was a degradation in asphalt binder’s fatigue resistance after the addition of waste toner [14]. Notani and Mokhtarnejad indicated that the asphalt mixture using 8% WT improved both the wetting and molecular diffusion processes of self-healing capability [15]. Huang et al. studied the effect of waste toner on the workability, rutting, and moisture-induced damage properties of asphalt. It was found that using waster toner as a modifier makes asphalt binder stiffer, decreasing moisture susceptibility and pumping capability. Furthermore, the workability and moisture resistance of the MT-modified binder was reduced, whereas the asphalt mixtures’ rutting resistance properties increased [16].

Waste-toner-based TonerPave was developed to reduce pavement rutting and cracking in Australia. Further, Lombardo reported that utilizing TonerPave in the asphalt mixtures reduced the use of asphalt and fine aggregates, which is economically beneficial in road construction [17].

In summary, although limited literature demonstrated the potential benefits of using WT as a modifier to improve the mechanical properties of asphalt binder, their results were contradictory. Some potential negative impacts on base asphalt were also noticed, which may be due to the variety of material sources used in previous studies. Therefore, more research on different sources of waste toner is still greatly needed.

### 1.2. Research Objectives

The objective of this research was to investigate the potential benefit of waste toner in the civil engineering area. In this regard, the specific objectives are as follows:

(1) To evaluate the chemical composition and the modification mechanism of waste toner binders.
(2) To investigate the optimum content of the recycled ink and best application when added to base asphalt.
(3) To investigate the influence of waste toner source and particle size on the rutting potential of the modified asphalt binders.
(4) To determine the performance grade of asphalt modified with different types of waste toner.

## 2. Materials

### 2.1. Asphalt Binder

In this study, Pen70# (according to Chinese standards) was chosen as base asphalt, which was collected from the CCIC inspection company in Jiangsu province (China). The main physical properties of the base asphalt are listed in Table 1.

### 2.2. Waste Toner Modifier (WTM)

**Figure 1:**
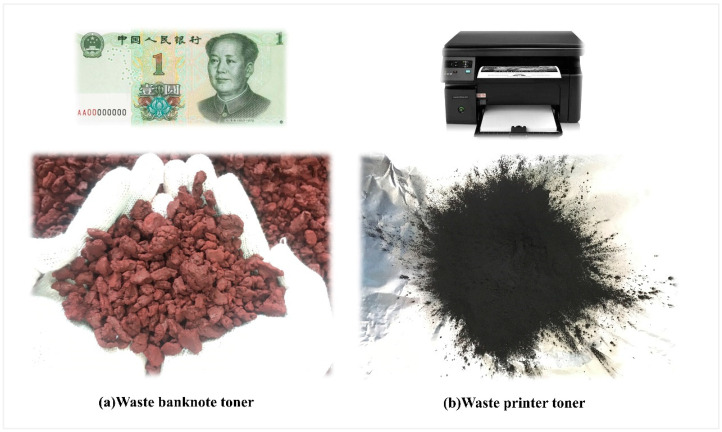
Appearance of waste toner modifiers.

In this study, two types of waste toners from different sources referred to as WBT and WPT were used to prepare toner-modified asphalt (TMA) binders. WBT was a recycled toner used to produce Chinese currency (Renminbi) supplied by a banknote printing company located in the city of Shanghai, China. WPT was a normal printer toner typically used in office printer machines. The appearance of different waste toner modifiers is shown in Figure 1.

## 3. Experimental Program

**Figure 2:**
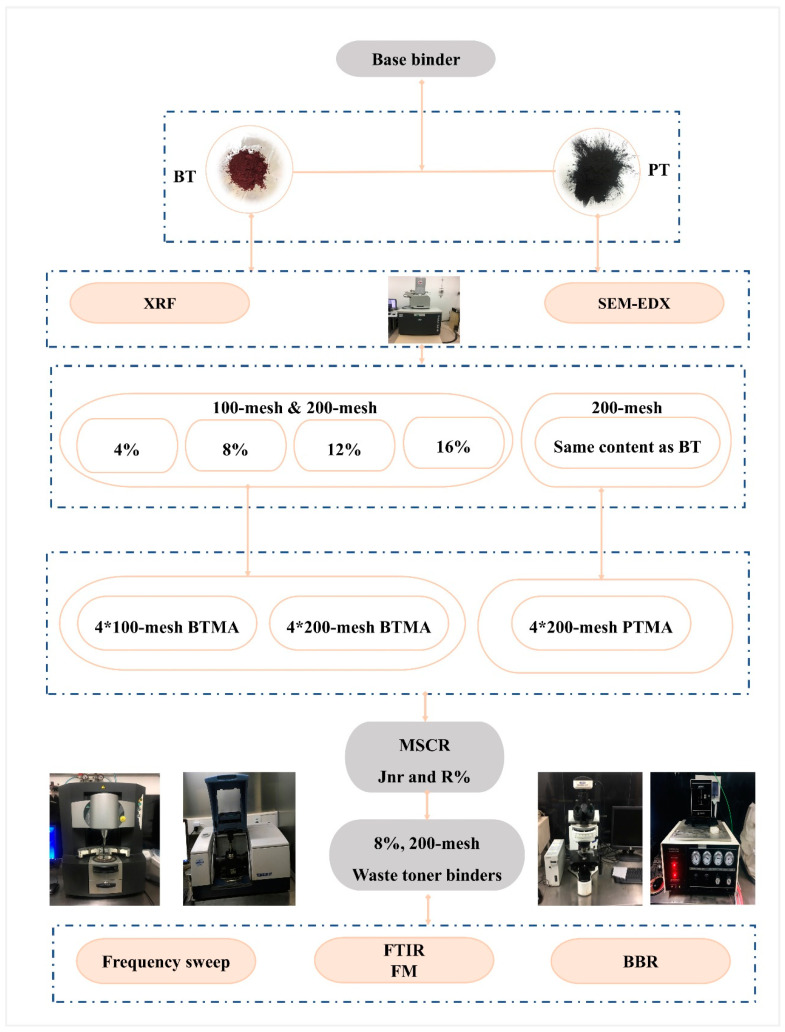
Flowchart of experimental design.

To focus on the effect of toner material, one type of binder was used in this study. It was mixed with banknote toner (BT) and ordinary printer toner (PT) at a weight ratio of 4%, 8%, 12%, and 16% and particle size of 100-mesh (0.150 mm) and 200-mesh (0.075 mm) to produce the modified asphalt binders. Rheology tests such as dynamic shear rheometer (DSR) and bending beam rheometer (BBR), as well as microscopic tests were performed. For each test used in this study, two replicates of each binder were prepared. The detailed flowchart of the experimental design is shown in Figure 2.

### 3.1. Toner Asphalt Preparation

**Figure 3:**
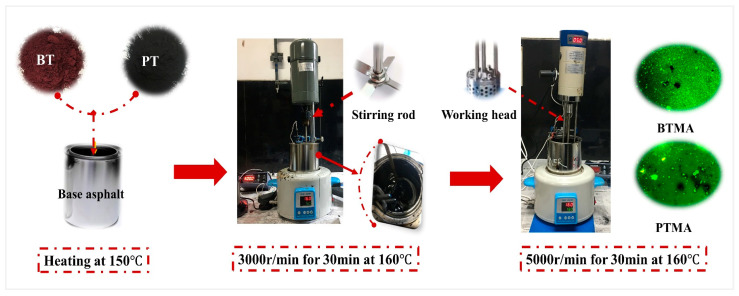
Toner-modified asphalt binder production process.

The wet process was used to produce waste toner-modified asphalt binder. The method of preparing the modified asphalt binder is summarized in Figure 3.

The modified-asphalt binders with WBT and WPT at a content of 4%, 8%, 12%, and 16% were produced using both a high-speed mixer and a high-speed shearing machine. The mixing condition (temperature, time, and shear velocity) and waste toner contents were determined based on the previous research conducted by Rondon-Quintana et al. [18]. The following are the steps of the preparation procedure of toner modified asphalt binders:

(1) One type of PEN70 binder was heated at the temperature of 150 °C to achieve the liquid state.
(2) Four contents (4%, 8%, 12%, and 16%) and two particle sizes (100-mesh and 200 mesh) of waste toners were added in batches into base asphalt at the same temperature. Manual stirring until no visible particles of toner could be seen was performed for 10 min. After this, a high-speed mixer machine was used for continuous mixing with a speed of 3000 r/min at 160 °C for 30 min.
(3) A high-speed shearing machine with a speed of 5000 r/min was performed at 160 °C for 30 min.

### 3.2. Microscopic Characteristics of Waste Toners and Waste-Toner-Modified Asphalt Binders

Chemical composition quantifying of waste toners was carried out by scanning electron microscopy with energy dispersive X-ray (SEM-EDS) using an FEI Quanta 200 FEG instrument.

The concentration of chemical elements present in waste toners was determined by X-ray fluorescence (XRF) using the XR-1800 instrument.

In order to identify the chemical functional groups of waste toner binders and to analyze the changes in the molecular structure of base binders after adding toners, the FTIR test was performed using Bruker TEN-SORFIR spectrometer. The FTIR spectrum in the range of 4000–500 cm^−1^ with a 4 cm^−1^ resolution was recorded. 

The fluorescence microscopy (FM) test is commonly used to observe the fluorescence properties and the dispersion of modifiers in asphalt binders. In this research, the fluorescence effect and the dispersion of waste toner particles were analyzed using an FM Olympus instrument.

### 3.3. Rheological Properties of Waste-Toner-Modified Asphalt Binder

#### 3.3.1. Frequency Sweep Test

A dynamic shear rheometer (DSR) is commonly used to investigate the properties of asphalt binder at intermediate to high temperatures. In this study, DSR was used to perform frequency sweep tests with frequency ranges from 0.01 to 20 Hz. The parallel plates of 25 mm diameter with a 1 mm testing gap were used at 50 °C and 60 °C, and 8 mm diameter plates with a 2 mm testing gap were used at 30 °C and 40 °C, according to AASHTO 315 [19]. Based on the time–temperature superposition principle (TTSP), the master curves of complex modulus and phase angle of base asphalt and waste toner-modified asphalt binders were constructed by a sigmoidal model and double logistic model [20] at the reference temperature of 40 °C.

#### 3.3.2. Multiple Stress Creep Recovery (MSCR) Test

The non-recoverable creep compliance (*J_nr_*) and deformation recovery percent (R%) have been used by numerous studies as a standard testing method to evaluate modified asphalt binders’ high-temperature performance [21,22,23,24,25].

In addition to these two parameters, the percent difference in *J_nr_* (*J_nr_*-diff) at 0.1 kPa and 3.2 kPa has been used to indicate the stress sensitivity of asphalt binder. Thus, *J_nr_*, R%, and *J_nr_*-diff (for only rolling thin film oven (RTFO) aged samples) were used in this study to investigate the high-temperature performance of waste-toner-modified asphalt at 64 °C under 0.1 kPa and 3.2 kPa stress levels according to ASTM D7405. For each binder, two replicates were prepared and tested.

#### 3.3.3. Continuous Grading Temperatures (High and Low Temperature)

The continuous grading temperatures of waste-toner-modified asphalt binders were determined according to ASTM D7643-16 [26]. Base asphalt and waste-toner-modified asphalt binders were subjected to shear oscillation via a dynamic shear rheometer (DSR) based on AASHTO315 [27]. After obtaining G*/sinδ of asphalt binders before and after RTFOT at two temperatures for each of them, continuous grading at high temperatures was determined.

The base asphalt and waste toner asphalt binders aged by means of RTFOT were further subjected to accelerated aging using a pressurized aging vessel (PAV), in accordance with AASHTO R28 [28]. The PAV aged base asphalt and toner asphalt binder samples were tested in order to determine m-value and stiffness by using a bending beam rheometer (BBR), following AASHTO T313 [29]. Then, after measuring m-values and stiffness at two different low temperatures, continuous grading low temperatures were determined.

## 4. Results and Discussions

### 4.1. Microscopic Characterization of WTs and TMAs

#### 4.1.1. SEM-EDS and XRF

**Figure 4:**
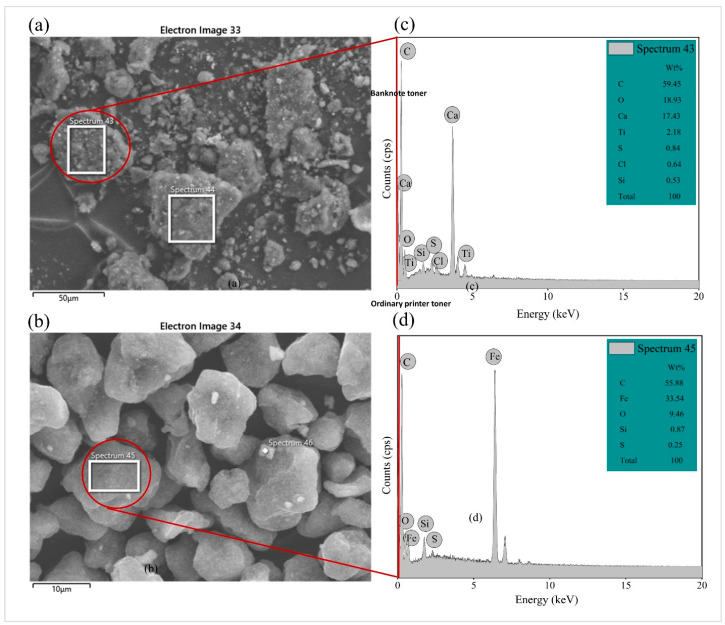
SEM-EDS of the waste toner powders. (**a**) SEM of WBT; (**b**) SEM of WPT; (**c**) EDS of WBT; (**d**) EDS of WPT.

The morphology and chemical composition of waste toners were investigated through scanning electron microscopy (SEM) coupled with energy-dispersive X-ray spectroscopy (EDS). Figure 4a,b show the SEM images of waste toner powders. Waste banknote toner powder (Figure 4a) showed particles with cubic shapes, whereas the waste printing toner powder showed some particles with rough edges. Figure 4c,d show the EDS analysis of the waste banknote toner (WBT) and that of the waste printer toner (WPT). The EDS analysis of the waste banknote toner revealed the presence of C, O, Ca, Ti, S, Cl, and Si elements. The major components contributing to WBT were C, O, and Ca. The EDS analysis revealed that the WPT powder consists mainly of C, Fe, and O, whereas the minor components were Si and S. Both WBT and WPT have C, O, Si, and S elements in them.

In XRF analysis, the concentrations of different chemical elements present in waste toner powders (presented as oxides) were determined. The composition of different elements present in WBT and WPT are tabulated (Table 2). XRF analysis reveals that Ca and C are the major elements present in WBT powder, with CaO and CO_2_ phases accounting for 44.15% and 32.15% of WBT, respectively. Moreover, the XRF analysis of ordinary printer toner powder reveals a high iron oxide (Fe_2_O_3_) content accounting for 92.08 of it. The XRF analysis shows the abundant presence of Ca and C for WBT and Fe for WPT, which can be confirmed by the EDS results.

The ordinary printer waste toner could be classified as a more magnetite toner compared to banknote toner due to the large amount of iron oxide component present in it [3]. Further, the adhesion performance of waste toner with base binder could be investigated by an alkalinity approach as per [30]. The alkalinity (M) response is given by the ration of calcium oxide (CaO) content and the sum of silicon dioxide (SiO_2_) and phosphorus pentoxide (P_2_O_5_) content, Equation (1).

(1) $$M=\frac{W(CaO)}{W(S_{i}O_{2})+W(P_{2}O_{5})}$$

The WBT has an alkalinity of 0.28, whereas the WPT could not be determined due to the lack of calcium oxide compound. Therefore, the waste toner materials were classified as low-alkalinity materials. This affirms that waste toner material may be unsuitable for promoting effective adhesion with asphalt binder.

#### 4.1.2. Fourier Transform Infrared Spectroscope (FTIR) Test

Commonly functional groups found in waste toner powders include aromatic, acrylic resins, and so on. Table 3 shows several common functional groups with corresponding peaks in waste toner powders [4,31,32,33].

**Figure 5:**
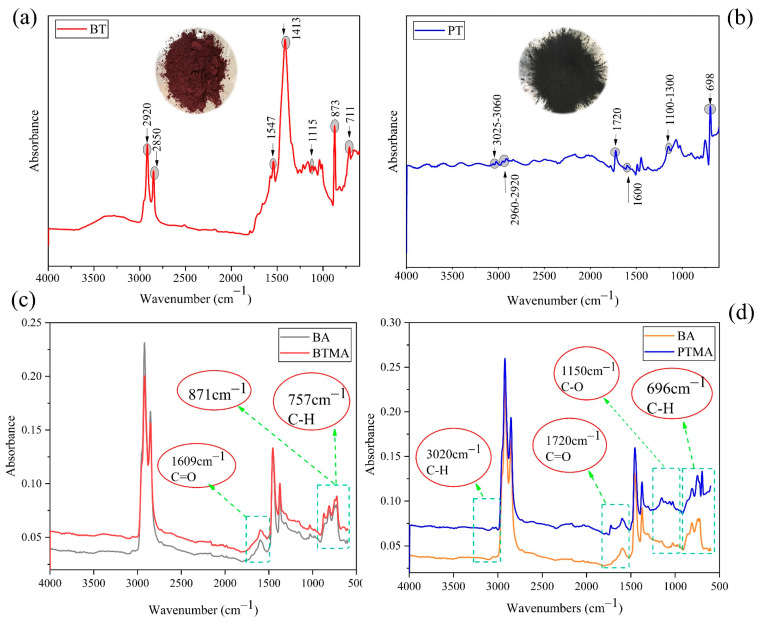
ATR-FTIR spectrum of (**a**) BT, (**b**) PT, (**c**) BTMA, and (**d**) PTMA.

The FT-IR spectra of BT, PT, BTMA, and PTMA are displayed in Figure 5a–d, respectively. The results indicated that both BTMA and PTMA have different absorption bands, which demonstrates that these two types of waste toners are different in their molecular structure. As Figure 4c shows, the remarkable peaks were located at a wavenumber of 757 cm^−1^, 871 cm^−1,^ and 1609 cm^−1^ for the banknote toner used in this study. The peak at 757 cm^−1^ was caused by the bending vibration of the C−H bond, whereas the peak at 871 cm^−1^ still unknown. The peak at 1609 cm^−1^ was an existing peak in which the intensity slightly changed after WBT modification. This might be caused by the stretching vibration of aromatic C=C. Additionally, as seen in Figure 4d, these peaks at 3020 cm^−1^, 1720 cm^−1^, 1150 cm^−1,^ and 696 cm^−1^ appeared at printer toner. The weak peaks around 3020 cm^−1^ and 1150 cm^−1^ are ascribed to the stretching of aromatic group C−H and the ester group C−O, respectively. Furthermore, the peak at 1720 cm^−1^ originates from the carbonyl stretch, whereas the peak around 696 cm^−1^ results from the aromatic ring bending.

Based on the FTIR analysis results of both BTMA and PTMA, there were new functional groups generated when WBT and WPT were added to the base asphalt. Therefore, there was a chemical reaction between the waste toner (WBT and WPT) materials and the base asphalt binder.

#### 4.1.3. FM Analysis

**Figure 6:**
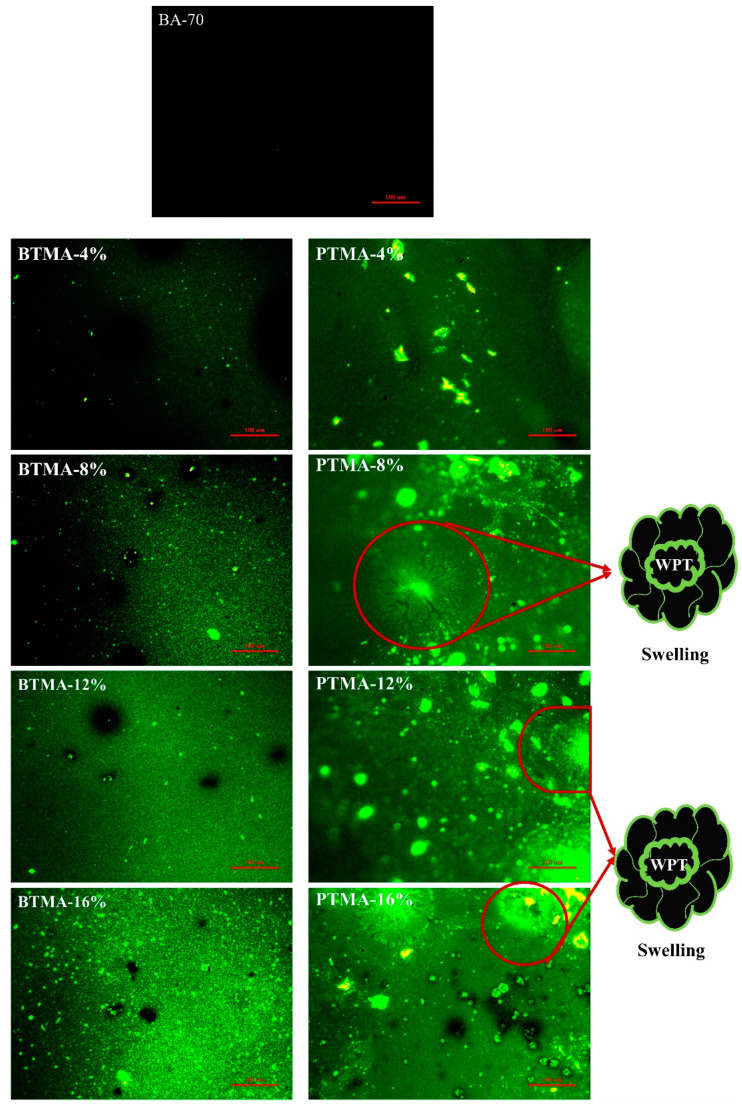
Fluorescence micrograph of all binders.

The dispersion of waste toner powders in asphalt binder was further investigated by FM and the results were illustrated in Figure 6. Compared to the micrograph of base asphalt binder, both BTMA and PTMA showed bright spots, indicating the strong fluorescent property of waste toner modifiers. As shown in Figure 6, 4%WBT dispersed in asphalt binder in the form of finer spherical shapes, whereas 4%WPT reveals an agglomeration behavior between printer toner powders and asphalt binder, which could be explained by the poor compatibility between them during the modified asphalt production. However, WPT was uniformly dispersed in the asphalt phase. When 8% of WTB was added, the bright spots filled a large area of the asphalt phase, and gradually increased with the increase in the WBT content, which formed a semi-network structure. Furthermore, the swelling phenomenon could be observed when the content of WPT reached 8%, as shown in Figure 6 PTMA-8%. This may be attributed to the fact that the content of WPT was sufficient, which in turn could be beneficial for the improvement of the rheological properties of asphalt binder.

### 4.2. High-Temperature Rheological Property

#### 4.2.1. Influence of Waste Toner Particle Size and Content

**Figure 7:**
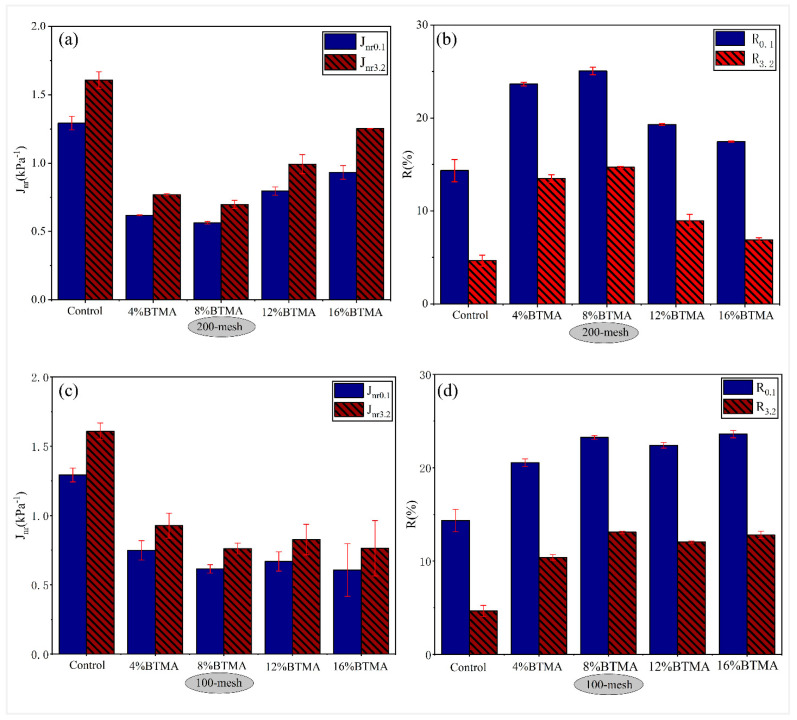
*J_nr_* and R for BTMAs with various toner particle sizes: (**a**) *J_nr_* 200-mesh, (**b**) R200-mesh, (**c**) *J_nr_* 100-mesh, and (**d**) R100-mesh.

**Figure 8:**
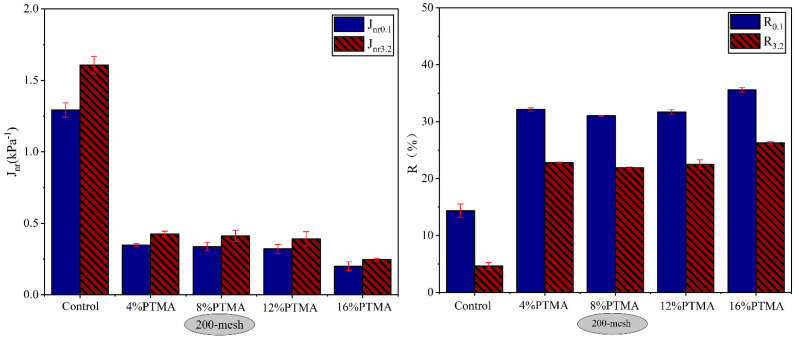
*J_nr_* and R for PTMAs with toner particle size of 200-mesh.

Figure 7 and Figure 8 summarized the $J_{nr}$ and R after 10 creep-recovery cycles for BTMAs and PTMAs with various toner particle sizes at 64 °C under stress levels of 100 Pa and 3200 Pa. When the $J_{nr}$ value is lower and the R value is higher, asphalt binder has good resistance to rutting. As illustrated in Figure 7 and Figure 8, different binders responded to creep-recovery cycles in completely different manners depending on the stress levels, waste toner source, and particle sizes as well as contents.

As shown in Figure 7a,c, the non-recoverable creep compliance (*J_nr_*) of 200-mesh BTMA and 100-mesh BTMA is significantly lower than that of base asphalt, indicating the improvement of elastic properties. At both stress levels of 0.1 kPa and 3.2 kPa, the *J_nr_* values of 200-mesh BTMAs were slightly lower than those of 100-mesh BTMAs, indicating BTMA with 200-mesh has a better rutting resistance compared to that with 100-mesh. In addition, combining the results of Figure 7 and Figure 8, the optimum toner content for BTMA and PTMA was determined to be 8%.

**Figure 9:**
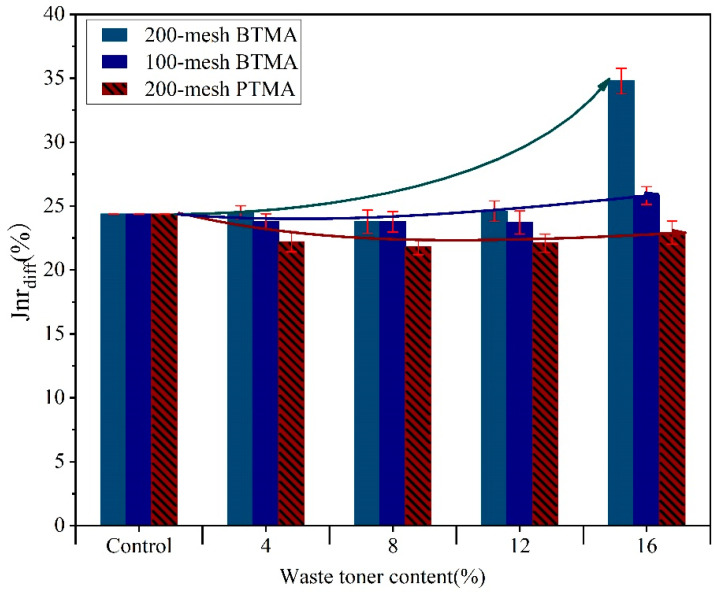
*J_nr_*-diff for all binders.

Figure 9 illustrated the results of *J_nr_*-diff for asphalt binders at 64 °C. All binders showed a *J_nr_*-diff percentage lower than 75%. Furthermore, the modified asphalt with WPT had lower *J_nr_*-diff than both the base asphalt and the BTMA, implying that modifying the asphalt binder with WPT can reduce the sensitivity of base asphalt to shear stress while also improving its stability to impact traffic loading.

#### 4.2.2. Continuous Grading High Temperature

**Figure 10:**
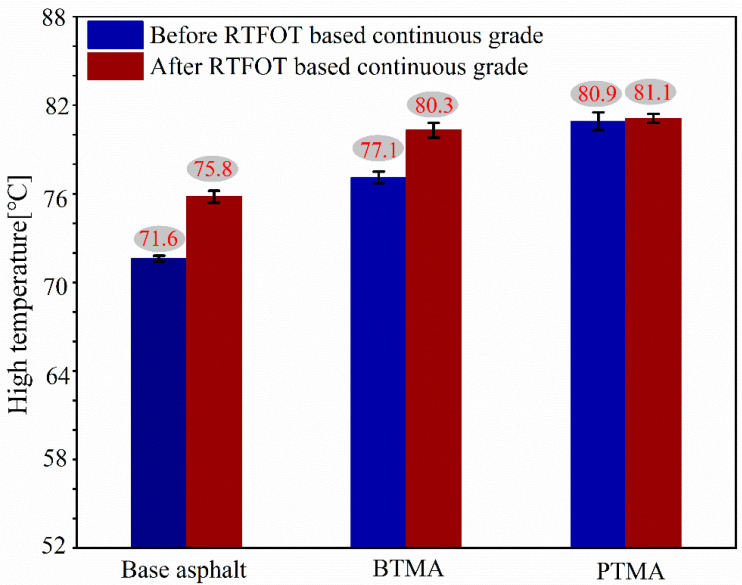
Continuous grading high temperature for all binders.

Figure 10 illustrates the continuous grading high temperatures for the binders, which are 71.6 °C for the base binder, 77.1 °C for BTMA, and 80.9 °C for PTMA, respectively. For all binders, the continuous grading high temperatures before aging were lower than those after aging. The effects of different sources of waste toner on the high temperature of base asphalt binder, as shown in Figure 10, indicate that base binder exhibits a lower high temperature than the waste-toner-modified asphalt binders (BTMA and PTMA). Moreover, PTMA shows a higher high temperature compared to BTMA. This could be explained by the strength of chemical reactions revealed based on the FTIR analysis.

### 4.3. Low-Temperature Rheological Property

#### 4.3.1. Continuous Low-Temperature Grading

**Figure 11:**
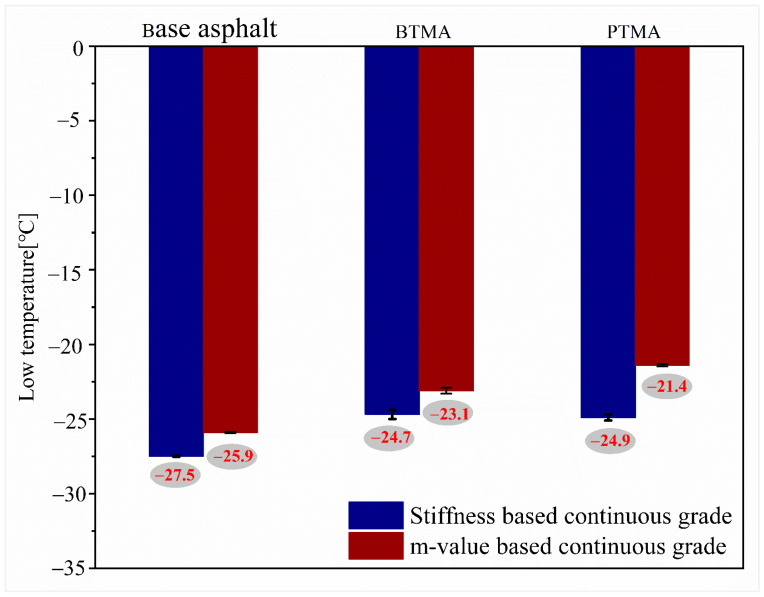
m-value and stiffness-based continuous grading low temperature for all binders.

Figure 11 shows both m-value and stiffness-based continuous low-temperature grading for base asphalt binder, BTMA, and PTMA. As can be seen, no matter whether based on m-value or stiffness properties, waste-toner-modified asphalt binders (BTMA and PTMA) show higher low temperatures compared to the base asphalt binder. In addition, BTMA shows comparable low temperature with PTMA based on stiffness criteria, whereas it shows a lower low-temperature grade than PTMA if m-value-based criteria are used. Overall, the continuous grading low temperatures are −25.9 for the base binder, and −23.1 and −21.4 for BTMA and PTMA, respectively.

#### 4.3.2. Low-Temperature Cracking Potential

**Figure 12:**
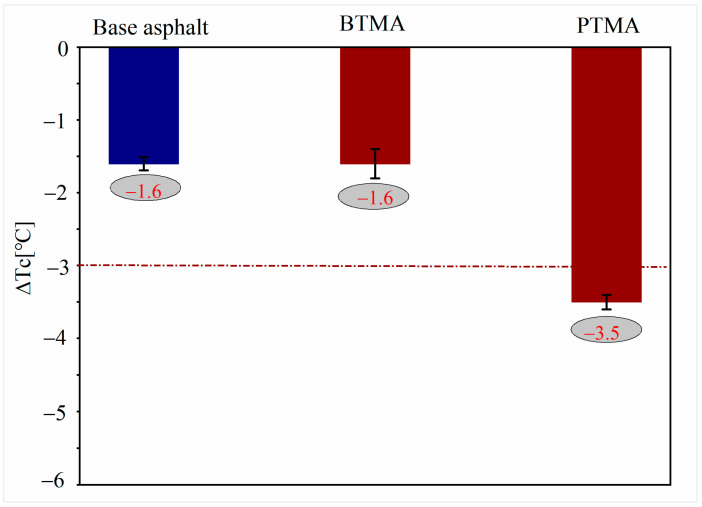
∆Tc for all binders.

The parameter ∆Tc is defined as the difference between the stiffness-based continuous low-temperature grading and the m-value-based continuous low-temperature grading obtained from the bending beam rheometer test. ${∆T}_{C}$ was used as an indication of the cracking susceptibility properties of asphalt binder [34,35]. Asphalt binder is considered to be more susceptible to cracking when the ∆Tc parameter reached the threshold value of −3 and −5 [36]. As shown in Figure 12, BTMA shows comparable ${∆T}_{C}$ value (−1.6) with the base asphalt binder, whereas PTMA has lower ${∆T}_{C}$ value (−3.5) than BTMA, and reached the cracking temperature of −3 °C, which implied that compared to BTMA, PTMA was more susceptible to cracking due to minimized durability after aging. In addition, statistical hypothesis analysis was also conducted using *t*-test to investigate the significance of adding waste toner modifiers into asphalt on the ${∆T}_{C}$ values. The results indicated that there is no significance difference between ${∆T}_{C}$ values of base asphalt and that of BTMA, whereas adding the WPT powders significantly decreased the ${∆T}_{C}$ values.

### 4.4. Complex Modulus Property from the Frequency Sweep Test

**Figure 13:**
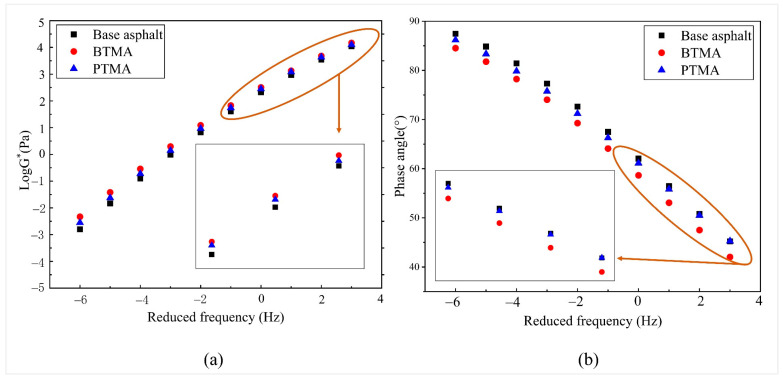
Frequency sweep for all binders at a reduced frequency: (**a**) complex modulus, (**b**) phase angle.

Frequency sweep tests were performed at the frequency ranges between 0.01 and 20 Hz to describe the linear viscoelastic responses of waste toner asphalt binders. Figure 13 presents the complex shear modulus (G*) and phase angle (δ) values of BTMA and PTMA regarding a reduced frequency at a reference temperature of 40 °C. The G* and δ values at low frequencies describe the pavement’s behavior at higher temperatures, whereas at high frequencies refer to the pavement’s behavior at lower temperatures.

As shown in Figure 13a, the addition of waste toners improved the complex shear modulus values of both BTMA and PTMA. In addition, the complex moduli of waste-toner-modified asphalt were improved across all frequency ranges, which indicated that using such material as an asphalt modifier reduced the temperature susceptibility of the asphalt binder. Furthermore, as illustrated in Figure 13a, at low frequencies (i.e., high temperatures), the G* value of PTMA was slightly lower than that of BTMA. Figure 13b shows that as the frequency increases, the phase angle decreases for both BTMA and PTMA. Additionally, the phase angle value of base asphalt binder decreased after waste toner powders were added, implying that the elasticity of waster-toner-modified asphalt is critical when exposed to loading. It was also observed that BTMA has a lower phase angle than PTMA.

## 5. Summary and Conclusions

This paper investigated the chemical and rheological properties of waste-toner-modified binders. WT binders were produced in the laboratory, using two sizes for toner WBT and one size for toner WPT, and four waste toner dosages. A series of rheological and microscopic tests were performed on both base binders and modified binders. Based on the experimental results, some findings and conclusions were drawn:

- The XRF results reveal that CaO and Fe_2_O_3_ are the main components of both WBT and WPT. Based on FT-IR results, BTMA and PTMA have different molecular structures. Furthermore, a chemical reaction occurred between waste toners and asphalt binders.
- Based on MSCR (J_nr_ and R) tests, the waste-toner-modified asphalt binder showed better rutting resistance than the base asphalt binder. Moreover, in terms of waste toner powder particle size, the 200-mesh was preferable. An amount of 8%WT was sufficient to produce a toner-modified asphalt product with an acceptable low-temperature property and a greater high-temperature property.
- From the frequency sweep test, it was found that BTMA and PTMA have better elastic properties than base asphalt. In particular, BTMA exhibited greater elastic properties compared to PTMA.
- The ΔTc parameter indicated that PTMA has lower resistance against non-load-related cracking than BTMA.
- Waste toner can enhance the high-temperature performance of base asphalt binder due to the chemical reaction occurred during the production process. The addition of 8% toner powder changed the continuous grade of base asphalt from PG71.6-25.9 to PG80.3-23.1 for banknote toner, and from PG71.6-25.9 to PG81.1-21.4 for printer toner. The rheological properties of waste-toner-modified asphalt could be affected by the source, quantity, and particle size of the waste toner.

The application of waste printing ink in pavement construction could be a viable approach for recycling such material. However, more research is needed to fully understand the low-temperature properties of waste-toner-modified asphalt, as well as its compatibility properties.

## Figures and Tables

**Table 1:** The physical property of base asphalt.

Test	Unit	Test Result	Method
Ductility at 15 °C	cm	>100	T0605-2011
Penetration (25 °C, 5 s, 100 g)	0.1 mm	68	T0604-2011
Softening point	°C	50.0	T0606-2011
Flash point	%	>300	T0611-2011

**Table 2:** Components of waste toner.

WBT		WPT	
Compound	Content (%)	Compound	Content (%)
CaO	44.1537	Fe_2_O_3_	92.0872
CO_2_	32.1581	SiO_2_	4.9261
Ti0_2_	6.7877	SrO	1.3260
Fe_2_O_3_	4.4468	SO_3_	0.6171
SO_3_	2.7603	Ti0_2_	0.5503
ZrO_2_	2.2237	MnO	0.4146
BaO	1.6514	ZnO	0.0787
SiO_2_	1.4844	——	——
P_2_O_5_	0.0514	——	——
Other oxides	4.2824	Other oxides	——

**Table 3:** Common absorption peaks of waste toner.

Absorption Peak	Functional Group	Indication
3025–3060 cm^−1^	C−H	Stretching of aromatic group
2995 cm^−1^	C−H	CH_3_ group
2960–2920 cm^−1^	C−H	Stretching of CH_3_ and CH_2_ groups
2900 cm^−1^	C−H	Stretching of aliphatic (CH_2_ groups)
2918 cm^−1^	C−H	Stretching vibration
1733 cm^−1^		Carbonyl group of ester bond
1720 cm^−1^	C=O	Carbonyl stretch
1716 cm^−1^	C=O	Stretching of acrylate group
1601 cm^−1^	C=C	Aromatic ring
1509 cm^−1^		Bisphenol A-based polymer
1452 cm^−1^	C−H	Bending vibration of CH_3_
		Group
1150 cm^−1^	C−O	Ester group
790–569 cm^−1^	C−H	Aromatic vibration mode
757 cm^−1^	C−H	Out-of-plane bending vibration
698 cm^−1^	C–H	Aromatic ring bending
583 cm^−1^	Fe-O	Stretching in tetrahedral sites

## Data Availability

All related data have been presented in this paper.

## Sample Availability

Samples of the materials and compounds used in this study are available from the authors.
